# Diagnostic Value of Imaging and Serological Biomarkers in Pulmonary Sarcoidosis

**DOI:** 10.3390/arm92030020

**Published:** 2024-04-28

**Authors:** Yuehong Li, Guopeng Xu

**Affiliations:** Department of Respiratory and Critical Care Medicine, The Affiliated Suzhou Hospital of Nanjing Medical University, Suzhou Municipal Hospital, Gusu School, Nanjing Medical University, Suzhou 215000, China; liyuehong@stu.njmu.edu.cn

**Keywords:** pulmonary sarcoidosis, diagnosis, computed tomography, enhance, biomarkers, summarize

## Abstract

**Highlights:**

**What are the main findings?**

- We reviewed the common imaging and serological biomarkers of sarcoidosis in recent years.
- We summarized the value of imaging and hematological indicators in the diagnosis and differential diagnosis of sarcoidosis.

**What are the implications of the main findings?**

- It is of great significance to understand the changes of biomarkers in sarcoidosis.
- It is very helpful for doctors to diagnose sarcoidosis clinically and can indicate disease activity and prognosis.

**Abstract:**

Sarcoidosis is a multisystem granulomatous disease of an unknown aetiology. It can exist in many organs. Pulmonary and intrathoracic lymph nodes are most commonly involved. Lung sarcoidosis is uncommon in Asia. However, due to the large population of our country and the development of bronchoscopy, percutaneous lung puncture, and other medical technologies, the number of pulmonary sarcoidosis patients is on the rise. Pulmonary sarcoidosis patients have no obvious symptoms in the early stage, and the clinical manifestations in the later stage may vary from person to person. Eventually, the disease progresses to life-threatening pulmonary fibrosis. Therefore, patients with pulmonary sarcoidosis should receive a timely diagnosis. In recent years, the imaging features and serologic biomarkers of pulmonary sarcoidosis have been continuously studied. The diagnostic value of imaging and serologic biomarkers for pulmonary sarcoidosis is summarized below.

## 1. Introduction

Sarcoidosis is a multisystem disease with unknown aetiology that was first described by Jonathan Hutchinson in 1869 and is characterized by the presence of noncaseating granulomas in the affected organs [1]. Granulomas can be detected in any organ, but the lungs and intrathoracic lymph nodes are most commonly affected [2]. Although little is known about the pathogenesis of sarcoidosis, it is considered to be the result of abnormal immune responses caused by complex interactions between host/genetic and environmental/infectious factors [3]. Its clinical manifestations vary from person to person. The general symptoms of sarcoidosis include fever, fatigue, weight loss, etc. The most commonly affected organs in sarcoidosis are the lungs and bilateral hilar lymph nodes, which are characterized by cough, expectoration, and dyspnea, followed by the skin (subcutaneous nodules, multiple erythema) and eyes (decreased vision), liver (hepatomegaly, abdominal pain, jaundice), spleen (splenomegaly), musculoskeletal system (joint pain), nervous system (facial paralysis, neuralgia), and heart (chest pain, palpitations, arrhythmia), and other organs throughout the body can also be affected [3]. And its prevalence varies significantly according to geography and race [4]. People of any age are susceptible to this disease, which usually begins in adults under 50 years old, with approximately 70% of cases occurring between 25 and 40 years old, and the second peak occurs in women over 50 years old [5]. The incidence of sarcoidosis, which is a rare disease, in Asian countries is 1.4 per 100,000 [6]. However, due to the large population in China, pulmonary sarcoidosis is not rare in clinical practice. With the progression of invasive diagnostic and treatment technologies such as endobronchial ultrasound, percutaneous lung puncture, and thoracoscopy, the diagnosis rate of pulmonary sarcoidosis has increased [7]. The diagnostic guidelines for sarcoidosis published by the American Thoracic Society in 2020 proposed that the diagnosis of sarcoidosis be based on the following three items: the presence of corresponding clinical symptoms; the discovery of noncaseating necrotizing granulomatous inflammation in ≥1 tissue sample; and the exclusion of other granulomatous diseases [8]. In view of the complexity of excluding other granulomatous diseases, the diagnosis of sarcoidosis remains a clinical challenge, and due to the diversity and occultness of the clinical manifestations of patients, there are still a considerable number of patients with sarcoidosis who are missed or misdiagnosed [9]. Sarcoidosis is often called the big imitator, and is considered to be a “chameleon” diagnosis because it can be disguised as another disease, and there is no reliable imaging feature or biomarker available for diagnosis or disease monitoring [10]. Therefore, it is urgent to find a method to assist in the diagnosis of sarcoidosis. This article reviews the information regarding the diagnostic value of imaging features and blood markers for the diagnosis of pulmonary sarcoidosis that have been discovered in recent years.

## 2. Diagnostic Value of Imaging in Pulmonary Sarcoidosis

### 2.1. Computed Tomography (CT)

Noncaseating necrotizing granuloma is the histopathological key to sarcoidosis and is the most important diagnostic clue for correctly defining sarcoidosis. However, biopsy is invasive, and interventional access to the mediastinum introduces inherent risks (such as infection and bleeding) to patients, as well as sampling errors [11]. If certain imaging features are highly correlated with sarcoidosis, biopsy can be avoided in some cases. Noncontrast chest scanning is a traditional imaging technique for detecting and classifying chest lesions. Pulmonary sarcoidosis is mainly characterized by bilateral hilar lymph nodes that are roughly symmetrically distributed, which are found in up to 90% of patients [12]. In the study of Wang Ren et al. on the differential diagnosis of mediastinal and hilar lymphadenopathy, the enlarged lymph nodes in patients with pulmonary sarcoidosis were mostly located in the mediastinum and both hilar regions (141/172, 81.98%), but there was no statistically significant difference between the locations of the lymph nodes and central lung cancer, lymphoma, or lymph tuberculosis; 2, 4, 5, 7, and 11R groups of lymph nodes were the main involved objects; and most of the lymph nodes were not fused (*p* < 0.05) [13]. In a study investigating CT spectral imaging of 21 patients with sarcoidosis and 39 patients with Hodgkin’s lymphoma, a monochromatic CT value of 40 keV in the arterial phase had the highest sensitivity (71.4%) and specificity (100%) in the differential diagnosis of pulmonary sarcoidosis and Hodgkin’s lymphoma, and there were statistically significant differences in the anatomical location, fusion, calcification, enhancement pattern, and enhancement degree of mediastinal enlarged lymph nodes [14].

### 2.2. Contrast-Enhanced Computed Tomography

Mediastinal lymphadenopathy can be caused by a variety of pathological conditions. Common malignant diseases include metastatic lymph nodes and malignant lymphoma, and benign causes include sarcoidosis and tuberculosis. Clinicians and radiologists must distinguish sarcoidosis from metastatic lymph nodes and lymphoma in the clinical environment for effective disease management and prognosis evaluation [15]. In a retrospective analysis of the diagnostic value of 64-slice spiral CT-enhanced scanning in pulmonary sarcoidosis, Tian Bin showed that 30 patients had obvious enhanced characteristics of enlarged lymph nodes, and the CT value increased by 32–43 HU after enhancement, with uniform lymph node density and clear boundaries without fusion. Also, intrapulmonary nodules were mostly seen in the middle and upper lungs, mostly distributed along the bronchovascular bundle, and reticular nodules with irregular shapes of 2–5 mm were the most common type of nodules [16]. These enhanced lymph node characteristics were basically the same as those in the study of He Yu et al. [17]. Because CT is noninvasive, easy to perform, and highly repeatable, it has obvious diagnostic value for patients with early pulmonary sarcoidosis. An analysis of the diagnostic effect of chest enhanced CT for pulmonary sarcoidosis revealed that the main manifestations of the intrathoracic lymph nodes were medium to high diffuse enhancement, with some necrosis, no fusion between lymph nodes, and no infiltrative changes. Enlarged hilar or mediastinal lymph nodes can easily cause compression or displacement of bronchi around the hilum in patients, and calcification of the lymph nodes is also one of the main manifestations [18]. Wang Ren et al. also suggested the same conclusion: lymph nodes in patients with pulmonary sarcoidosis showed roughly uniform enhancement after enhanced scanning, while the enhancement of lung cancer lymph nodes was not obvious. Also, in that study, tuberculous lymph nodes showed circular enhancement, and lymph nodes affected by lymphoma showed slight enhancement [16]. However, there was no obvious statistical significance in the difference in enhancement degree grading (*p* = 0.242), which could not be excluded due to the small sample size.

### 2.3. Fluorodeoxyglucose Positron Emission Tomography/Computed Tomography (FDG-PET/CT)

**Figure 1:**
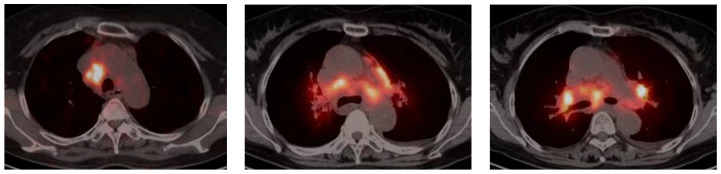
PET/CT images of pulmonary sarcoidosis.

FDG-PET CT is a combination of positron emission tomography (PET) and computed tomography (CT), and PET imaging is characterized by increased FDG uptake. Inflammatory cells in sarcoidosis consume glucose at a much higher level than surrounding non-inflammatory cells during the inflammatory process, resulting in increased glucose metabolism in the inflammatory lesions (Figure 1). The sensitivity of PET-indicated lesions is mainly related to the SUV value, while CT mainly depends on the size of the lesion. Therefore, for small lesions, PET has a higher sensitivity than CT [19]. We performed a retrospective collection of FDG-PET/CT images of patients undergoing spinal MRI and FDG-PET/CT scans and used the standard uptake value (SUV) to evaluate the FDG uptake of the lesion area. The results showed that the mean SUV of the spinal sarcoidosis group was 4.38, ranging from 3.30 to 4.93, which was significantly higher than the mean SUV of the control group (1.87, ranging from 1.42 to 2.74). Therefore, SUV values based on FDG-PET/CT images may help to distinguish spinal sarcoidosis from other non-inflammatory lesions [20].

The combination of PET/CT imaging metabolism and anatomy can more clearly show the boundary, shape, and relationship with the surrounding lesions; guide the clinical selection of appropriate biopsy sites; improve the diagnostic rate of tissue biopsy; and reduce the risk of complications. After treatment, PET/CT shows that the SUV of the lesions is significantly reduced. Therefore, this examination technique has important value for the diagnosis of sarcoidosis, the selection of biopsy sites, and the evaluation of treatment efficacy. Although FDG-PET/CT is a powerful tool, it is not the first-line imaging method for sarcoidosis, because FDG uptake is affected by a variety of factors, such as blood glucose level, cell proliferation rate, etc. The patient’s clinical manifestations, imaging features, and other laboratory test results need to be considered comprehensively when interpreting PET/CT findings [19].

### 2.4. Cardiac Magnetic Resonance Imaging (Cardiac MRI)

Cardiac MRI is a noninvasive imaging technique that does not expose patients to ionizing radiation, making it more attractive than other imaging techniques. With its excellent resolution and unique ability to image myocardial oedema, abnormal perfusion, and fibrosis, cardiac MRI has become an important tool for the diagnosis and management of cardiac sarcoidosis. Cardiac MRI imaging can be used to develop personalized treatment plans based on the different manifestations of each patient. Cardiac MRI can take images of the whole heart, mapping the structure and function of the myocardium. T2-weighted imaging can evaluate the degree of myocardial oedema, while late gadolinium enhancement (LGE) imaging can evaluate the presence of fibrosis [21]. In a study on MRI differentiating cardiac diseases, the results showed that the cardiac sarcoidosis group was mainly characterized by patchy 3-layer LGE (*p* = 0.01), while the myocardial infarction group was commonly characterized by confluent transmural LGE (*p* = 0.04) and vascular distribution (*p* < 0.001). Therefore, LGE helps to distinguish patients with cardiac sarcoidosis from those with recent myocardial infarction [22]. However, the presence of metal substances in the body may affect the scanning results. The interpretation of cardiac MRI results needs to be combined with the patient’s clinical manifestations and other laboratory findings; therefore, cardiac MRI results alone cannot confirm the diagnosis of sarcoidosis.

### 2.5. Brain Magnetic Resonance Imaging (Brain MRI)

Brain MRI is the first choice of imaging diagnostic technique for neurosarcoidosis. Its image features include leptomeningeal thickening, tumour-like lesions, cranial nerve enhancement, and hydrocephalus. Leptomeningeal thickening is one of the most common radiological features of neurosarcoidosis. When leptomeningeal involvement occurs, T1WI shows diffuse or nodular leptomeningeal thickening and enhancement. Brain parenchymal lesions may show multiple small, non-enhanced periventricular white matter or subcortical white matter hyperintensities on T2WI. MRI is also good at showing cranial nerve involvement, and may show unidirectional or bilateral cranial nerve thickening or enhancement on enhanced T1WI. The imaging features of neurosarcoidosis spinal cord lesions change according to the different sites of involvement: intraspinal lesions show high signal on T2WI and low signal on T1WI. Extraspinal lesions, especially with leptomeningeal involvement, usually show linear or small lesions of enhancement. However, histological diagnosis is still the gold standard at present, and MRI alone cannot determine the diagnosis of neurosarcoidosis [23]. The above imaging biomarkers are shown in Table 1.

## 3. Diagnostic Value of Serum Biomarkers for Pulmonary Sarcoidosis

### 3.1. Angiotensin-Converting Enzyme (ACE) and Interleukin-2 Receptor (sIL-2R)

Sarcoidosis is a multisystem inflammatory disease of unknown aetiology. At present, no biomarkers have been shown to be effective in confirming the diagnosis of sarcoidosis. The most commonly used biomarkers are serum and bronchoalveolar lavage fluid biomarkers, but they lack the necessary specificity and sensitivity. According to current studies, angiotensin-converting enzyme (ACE) and soluble interleukin-2 receptor (sIL-2R) are the most widely used serum biomarkers for sarcoidosis, although their sensitivity and specificity are not ideal. ACE is produced by epithelioid cells within nodular granulomas, and high levels of serum ACE are considered to reflect the burden of granulomatous inflammation [24]. An ACE level > 2 N is a highly likely diagnostic indicator, and the sensitivity of ACE is quite good, but an elevated level alone is not specific enough for diagnosis [25]. Moreover, elevated ACE levels may also occur in patients with other granulomatous diseases, such as liver disease, lymphoma, diabetes, and hyperthyroidism. In addition, widely used antihypertensive drugs, such as ACE inhibitors (ACEI), can reduce serum ACE levels, resulting in false negative results [26,27,28]. Therefore, the value of ACE levels as diagnostic or prognostic tools is still controversial. In a large sarcoidosis study, it was found that ACE activity could be measured at different serum dilutions to explore the effect of ACEI on ACE levels. With increasing dilution, the concentration of ACEI in serum gradually decreased, resulting in a gradual loss of the inhibitory effect. Ideally, significant dilution of serum could be used to simulate the states of patients without taking drugs, so that the levels of uninhibited enzyme activity in patients’ serum can be determined objectively without interrupting drug therapy. However, angiotensin receptor blockers (ARBs) do not affect ACE activity [29]. Similarly, sIL-2R can also be elevated in patients with haematological malignancies, autoimmune diseases, and idiopathic pulmonary fibrosis [30]. A study of patients with sarcoidosis treated with methotrexate showed that serum levels of angiotensin-converting enzyme (ACE) and soluble IL-2 receptor (sIL-2R) were reduced after 6 months of treatment and correlated with increased lung function [31]. To date, neither ACE nor sIL2R testing is recommended as a diagnostic test for patients with sarcoidosis, for initial evaluation, or for routine testing of patients during follow-up [32]. Other biomarkers, such as chitotriosidase, neopterin, and serum amyloid protein, are associated with sarcoidosis, but may be markers of extrathoracic involvement or prognostic value and are not accurate enough for diagnosis. Therefore, more work is needed to confirm their usefulness [15]. The ideal biomarker should not be associated with other diseases, but should be highly sensitive. In addition, the ideal biomarker should not be invasive to obtain and should be reproducible. Relatively speaking, our ideal biomarker should be simple and inexpensive and require no sophisticated technology.

### 3.2. Serum Amyloid A (SAA) and Chitotriosidase (CTO)

SAA is a non-specific acute-phase protein produced by the liver during the acute phase response. Macrophages produce high levels of SAA in inflammatory conditions, suggesting that elevated SAA levels are a clinical marker of inflammation. Abnormal expression of SAA is associated with increased risk or poor prognosis in many chronic diseases, including atherosclerotic cardiovascular disease and cancer [33]. Elevated SAA levels have been found in sarcoidosis and appear to be associated with decreased lung function [34]. However, its correlation with disease activity is not clearly established.

CTO is a highly sensitive biomarker of sarcoidosis secreted by activated macrophages and neutrophils. It can reflect the activity and severity of sarcoidosis granuloma burden. CTO levels are significantly increased in patients with sarcoidosis, which is helpful for patient follow-up and detection of disease recurrence. However, CTO levels are also increased in patients with asbestosis, non-sarcoidosis pulmonary fibrosis, and lung cancer [35]. Lower levels of CTO have been reported after treatment with steroids or immunosuppressants [36,37].

### 3.3. Bronchial Alveolar Lavage Fluid (BALF)

BALF examination is an important adjunctive means in the diagnosis of sarcoidosis. Studies have shown that the percentage of lymphocytes in BALF is significantly increased in patients with sarcoidosis (*p* < 0.001), and the percentage and count of BALF CD4+ are significantly higher than those in controls (*p* < 0.001, *p* < 0.004, respectively), while the percentage of CD8+ is significantly decreased (*p* < 0.007, *p* < 0.027, respectively), resulting in an increased BALF CD4+/CD8+ ratio (*p* < 0.007) [38]. Clinically, a CD4/CD8 ratio > 3.5 and lymphocytosis >15% support the diagnosis of sarcoidosis. However, not all patients with sarcoidosis show BALF lymphocytosis. It is currently considered that the CD4/CD8 ratio in BALF does not reflect the severity of the disease [24].

### 3.4. JAK/STAT Signaling Pathway

The JAK/STAT signalling pathway is an important signalling system in cells which is involved in regulating various biological processes such as cell growth, differentiation, and survival. This pathway is particularly important in immune regulation and inflammatory response, and is closely related to the occurrence and development of various diseases, including tumours, autoimmune diseases, and inflammatory diseases. JAK inhibitors can inhibit the above pathway, thereby treating various diseases caused by the dysfunction of functional proteins in the JAK-STAT pathway. At present, JAK inhibitors have been used to treat some autoimmune diseases, such as rheumatoid arthritis, psoriasis, and inflammatory bowel disease [39]. In the treatment of sarcoidosis, the JAK/STAT pathway may also be a potential therapeutic target. However, the research on the specific role of the JAK/STAT pathway and targeted therapy in sarcoidosis is still relatively limited. More research is needed to explore its potential value in the diagnosis and treatment of sarcoidosis.

### 3.5. Monocytes and Platelet/Lymphocyte Ratio (PLR)

Serum biomarkers should be the most focused area for researchers, as these markers are the least invasive and the most easily available [40]. Relevant studies have shown that the main manifestations of laboratory findings of sarcoidosis are decreased lymphocytes and increased inflammatory indicators, such as the erythrocyte sedimentation rate and C-reactive protein [41]. Neutrophil, lymphocyte, monocyte, and platelet counts are all indicators that can be easily obtained from peripheral blood cell counts and play important roles in determining inflammation. Macrophages play a key role in the formation of granulomas, and monocytes, as precursors of macrophages, are found in blood and can be considered biomarkers for sarcoidosis [25]. The platelet-to-lymphocyte ratio (PLR) is an inflammatory marker in immune-mediated, metabolic, prethrombotic, and neoplastic diseases [42]. In a study on the correlation between serum angiotensin-converting enzymes and immunoinflammatory indicators in patients with sarcoidosis, the clinical data of 274 patients with stage II sarcoidosis over a 10-year period were retrospectively analysed. The results showed that the proportion of monocytes and the platelet/lymphocyte ratio in the elevated ACE group were significantly greater than those in the normal ACE group (t values were −4.58 and −3.23, respectively, both *p* values < 0.05), and there were varying degrees of correlation between the ACE level and monocyte proportion and the platelet/lymphocyte ratio (r values were 0.32 and 0.13, respectively, both *p* values <0.05) [43]. ACE has been proven to be a diagnostic reference for sarcoidosis. The above studies showed that the ACE level was correlated with the proportion of monocytes and the platelet/lymphocyte count to varying degrees. In the respiratory system, PLR has diagnostic value for respiratory syncytial virus (RSV) infection in children under 2 years old [44] and influenza A virus infection in children [45] (all *p* < 0.001). Among children under 2 years of age, the median PLR was 72.17 (49.63–108.26) in the RSV-positive group and 119.31 (90.32–223.07) in the RSV-negative group. The results showed that the PLR level in the RSV-positive group was significantly lower than that in the RSV-negative group (*p* < 0.001). When the PLR level was below 73, the specificity for diagnosing RSV infection in children was more than 90%. However, PLR levels were increased in patients with sarcoidosis. Studies on influenza A virus infection in children showed that PLR levels were also increased, and the cut-off value was 124, with sensitivity and specificity of 56.70% and 89.60%, respectively, while the cut-off value of PLR for predicting sarcoidosis was 158 [46].

### 3.6. Lymphocyte/Monocyte Ratio (LMR) and Neutrophil/Lymphocyte Ratio (NLR)

The lymphocyte-to-monocyte ratio (LMR) is a relatively new inflammatory marker that may reflect systemic inflammation, and it is used to evaluate the degree and prognosis of inflammation in autoimmune, cardiovascular, and pulmonary diseases, as well as malignant tumours. However, a retrospective analysis by Tugce et al. revealed that recurrence and parenchymal fibrosis were not related to the metabolic parameter LMR [47], and the LMR alone cannot predict the prognosis of patients with sarcoidosis, possibly due to the small number of patients studied and the lack of homogeneity between the groups.

The neutrophil-to-lymphocyte ratio (NLR) is a very inexpensive and easy-to-perform test, and is used as an indicator of inflammation in the lungs and for other malignant tumours. According to a systematic review and meta-analysis, studies have shown that the neutrophil-to-lymphocyte ratio (NLR) can be used for the diagnosis of acute appendicitis and the assessment of the severity of the disease. NLR > 4.7 was identified as a predictor of acute appendicitis, with a sensitivity of 88.89%, a specificity of 90.91%, and an AUC of 0.96 (*p* < 0.0001) [48]. However, in the study of Celalettin Korkmaz et al., it was found that the NLR level in patients with sarcoidosis (3.26 ± 2.13) was significantly higher than that in the control group (2.45 ± 2.41), with a cut-off value of 2.07 (*p* < 0.001) [49].

The significance of the NLR in the diagnosis, follow-up, and activity of sarcoidosis is not very clear. The use of the neutrophil-to-lymphocyte ratio for the differential diagnosis and prognosis evaluation of pulmonary sarcoidosis patients has been recommended in only a few studies. A study by Gungor et al. with a large sample size showed that, when the NLR was ≥2 (*p* = 0.031), it contributed to the diagnosis and prognosis of pulmonary sarcoidosis, but there was no statistically significant difference in the subgroup with sarcoidosis [50]. In addition, in another study demonstrating the relationship between sarcoidosis and the NLR, the correlation between ACE and the NLR was determined, which coincided with the findings of Gungor’s study [51]. Huang Guohua et al.’s study on the diagnostic value of the NLR in pulmonary sarcoidosis revealed that the serum NLR was significantly greater in patients with pulmonary sarcoidosis than in patients with pulmonary tuberculosis. The results of the ROC curve suggested that the optimal critical value of the NLR for excluding pulmonary tuberculosis was 2.203; that is, with an NLR ≥ 2.203 as the diagnostic standard for pulmonary sarcoidosis, the sensitivity was 85.48%, the specificity was 69.70%, the positive predictive value was 72.60%, and the negative predictive value was 83.64%, suggesting that the NLR has certain value in the diagnosis of pulmonary sarcoidosis and its differential diagnosis from pulmonary tuberculosis [52].

In a study on the relationship between haematological parameters and treatment response in patients with sarcoidosis, observations were made in 75 patients, of whom 42 (56%) received medications such as methylprednisolone, methotrexate, infliximab, topical steroids, or nonsteroidal anti-inflammatory drugs. The results showed progression in three patients. However, no significant differences were observed in NLR or PLR results between patients with treatment response and those without improvement (*p* = 0.39, *p* = 0.51, respectively) [49].

### 3.7. Serum Calcium Level

Hypercalcaemia is caused by increased bone resorption, decreased renal calcium clearance, and increased gastrointestinal calcium uptake. The parathyroid hormone (PTH) and vitamin D regulate bone mobilization, renal excretion, and intestinal calcium uptake to maintain calcium homeostasis. About 90% of patients with hypercalcaemia have primary hyperparathyroidism or malignant tumours. Other causes of hypercalcaemia include granulomatous diseases (such as sarcoidosis), genetic diseases, medications (such as thiazide diuretics), etc. Therefore, hypercalcaemia is not a specific manifestation of sarcoidosis, and other possible causes need to be investigated. In primary hyperparathyroidism, the level of parathyroid hormone (PTH) is elevated. Measuring the 24-h urinary calcium level can help to assess the kidney’s ability to process calcium. Elevated serum alkaline phosphatase (ALP) levels may be associated with abnormal bone metabolism. The joint detection of these indicators can be used to identify the cause of hypercalcaemia [53].

Changes in calcium metabolism are very common in patients with sarcoidosis, with hypercalciuria being associated with a persistent clinical phenotype and more active disease. There are no data on the specificity of calcium metabolic parameters as biomarkers for distinguishing different chronic interstitial lung diseases. An analysis of calcium metabolism in a cohort of 237 sarcoidosis patients in Italy confirmed that serum and urinary calcium concentrations were greater in patients with pulmonary sarcoidosis than in patients with idiopathic pulmonary fibrosis and chronic hypersensitivity pneumonia (*p* = 0.0004 and *p* < 0.0001). According to receiver operating characteristic (ROC) curve analysis, urinary calcium was more accurate in the differential diagnosis of nodular and nonnodular pulmonary fibrosis (AUC 0.7658 vs. 0.6205; *p* = 0.0026 vs. *p* = 0.1820), but there were no significant differences in serum or urinary calcium metabolic parameters between different clinical phenotypes and radiological stages in the sarcoidosis group [54]. This finding supports the hypothesis that calcium metabolism disorders may be a specific feature of nodular granulomas, suggesting that the assessment of calcium metabolism may be useful in the diagnosis of pulmonary sarcoidosis. In a review by F. Jeny et al., hypercalcaemia with normal or low 25-hydroxyvitamin D was also suggestive of sarcoidosis [25]. The above serological biomarkers are shown in Table 2.

## 4. Conclusions

Sarcoidosis is an idiopathic, multisystem disease that involves the formation of noncaseating epithelioid cell nodules in affected tissues or organs. Patients with pulmonary sarcoidosis may have no obvious clinical manifestations in the early stage, but with the progression of the disease, a poor prognostic indicator such as pulmonary fibrosis may occur. The disease trends towards younger patients and more females. If the disease cannot be diagnosed in time, it will seriously affect the quality of life and physical health of patients. With the progress of diagnosis and treatment technology, the diagnosis rate of pulmonary sarcoidosis is gradually increasing through invasive operations such as bronchoscopy, percutaneous lung puncture, and mediastinoscopy. However, these invasive operations inevitably lead to complications. CT, which can differentiate sarcoidosis from other mediastinal abnormalities according to mediastinal enlarged lymph nodes, is the preferred imaging method for the evaluation of pulmonary sarcoidosis. If conventional CT scans cannot detect patients with pulmonary sarcoidosis with typical morphological characteristics, dynamic contrast enhancement can effectively assist in the differential diagnosis. Previous studies have explored the important role of CT in the diagnosis of sarcoidosis and Hodgkin lymphoma based on mediastinal enlarged lymph nodes. At present, one of the problems in the study of sarcoidosis is the lack of a clear pathogenesis, specific biomarkers, and treatment options. Although many biomarkers have been evaluated in patients with pulmonary sarcoidosis in recent decades, they have not been established as the gold standard for diagnosing or predicting disease progression. The NLR is a marker that can be easily calculated from a routine blood cell count without additional work or cost, and is highly reproducible. Many studies have highlighted its success in predicting prognosis; however, more research is needed to determine its beneficial role in diagnosis.

These results are interesting; however, given that most of the studies were retrospective analyses, the retrospective design of these studies makes them inherently prone to referral and reporting biases, which may significantly affect the analysis and interpretation of the data. Also, their sample size was small; therefore, further studies involving large prospective cohorts are warranted. Future studies should focus on combining serum or BALF biomarkers with further refined imaging techniques, such as CT and MRI, to achieve the best cost-effectiveness ratio for the diagnosis of patients with pulmonary sarcoidosis.

## Figures and Tables

**Table 1:** Imaging biomarkers of sarcoidosis.

Biomarker	Lesion	Results	*p*	Control Group
Spectral CT	Pulmonary sarcoidosis	cut-off value: 40 keV in AP (sensitivity:71.4%, specificity:100%)	<0.001	Hodgkin’s lymphoma
Contrast-Enhanced CT	Mediastinal and hilar lymph nodes	no difference in lymph node reinforcement grade	0.242	Central lung cancer, lymphoma, lymphatic tuberculosis, giant lymph node hyperplasia, metastatic lymph node cancer
FDG-PET CT	Spinal cord sarcoidosis	mean SUV: 4.38 (3.30–4.93) higher than 1.87 (1.42–2.74)	0.02	Myelomalacia
Cardiac MRI	Cardiac sarcoidosis	cardiac sarcoidosis: patchy 3-layer LGE, myocardial infarction: confluent transmural LGE and vascular distribution	<0.001	Recent myocardial infarctions
Brain MRI	brain sarcoidosis	leptomeningeal thickening, cranial nerve enhancement, and hydrocephalus	-	-

**Table 2:** Serum biomarkers of sarcoidosis.

Biomarker	Lesion	Results	*p*	Control Group
ACE	sarcoidosis	mean ACE decreased with 17.2 U/L correlated with an increase in lung	<0.0001	sarcoidosis before 6 months of methotrexate treatment
sIL-2R	sarcoidosis	mean sIL-2R decreased with 1850 pg/mL with an increase in lung	<0.0001	sarcoidosis before 6 months of methotrexate treatment
SAA	sarcoidosis	50.231 ± 40.725 vs. 31.301 ± 37.375	<0.025	healthy volunteers
CTO	sarcoidosis	175.4 ± 89.4 vs. 34.2 ± 13.8	<0.0001	healthy volunteers
BALF	sarcoidosis	CD4+/CD8+ ratio rise	<0.007	healthy volunteers
JAK/STAT	sarcoidosis	the activity of JAK/STAT was present in 50% of patients	-	-
PLR	sarcoidosis	cut-off value: 158 (sensitivity:57%, specificity:93%)	<0.001	healthy volunteers
LMR	pulmonary sarcoidosis	relapse and parenchymal fibrosis were not associated with LMR	0.167	-
NLR	pulmonary sarcoidosis	cut-off value: 2.203 (sensitivity:85.48%, specificity:69.70%)	<0.001	tuberculosis
Serum Calcium Level	sarcoidosis	AUC 0.6195, 95% CI: 0.5090–0.7300	0.01756	idiopathic pulmonary fibrosis, chronic hypersensitivity pneumonitis
